# RT Box card for studying the control communication impacts on microgrid performance and stability

**DOI:** 10.1016/j.ohx.2022.e00322

**Published:** 2022-06-03

**Authors:** Maximiliano Aguilar, Sebastián Riffo, Antonio Veliz, Catalina González-Castaño, Carlos Restrepo

**Affiliations:** a Department of Electromechanics and Energy Conversion, Universidad de Talca, 3340000 Curicó, Chile; b Department of Engineering Sciences, Universidad Andres Bello, 7500971 Santiago, Chile

**Keywords:** Microgrid, Communication control card interface, Open-source, RT Box

## Abstract

This work presents the design process of an RT Box interface for control systems in microgrid applications. The control card allows implementing different controls and communication systems for microgrids in a physical environment, facilitating the development of robust control systems facing inherent adverse scenarios such as delays, loss of information packets, failures of partial or permanent communication and noise in signal overflows, among others. In addition, it permits the generation of programming resources and behavioral antecedents, helpful information for future users of the control card interface. The Plecs RT Box is a device that will enable a real-time simulation of different power electronics applications as can be a microgrid system. The built-in control card in this project is suitable as a complementary element of the RT Box, extending the capacity of this device to emulate a microgrid but testing real communication protocol between the microcontrollers that compose each of the distributed generation units (DGU). Tests were conducted to probe the communication protocols working correctly in a microgrid context, recreating real application scenarios.

**Specifications table**

**Table:** 

**Hardware name**	RT Box Microgrid Control Interface.	
**Subject area**	*Engineering and material science.*	
**Hardware type**	Electrical engineering and computer science.	
**Closest commercial analog**	HIL uGrid DSP Interface from Typhoon HIL.	
**Open source license**	CERN Open Hardware License (OHL)	
**Cost of hardware**	*300 USD*	
**Source file repository**	https://data.mendeley.com/datasets/mj6mx8d5c5/11	

## 1 Hardware in context

In recent years great concern has emerged about global warming, especially with fossil fuels to produce electricity. This concern has led to the investigation and use of renewable energies with a very low carbon footprint [1]. As of today, 2021, exponential developments and advances have been seen, especially in wind and solar energy [2], [3]. For example, in 2019, solar energy production reached 693 TWh and wind energy 1412 TWh [4]. These energies have been the most developed thanks to the manufacturing costs reduction and the development of power converters with high efficiency.

**Fig. 1:**
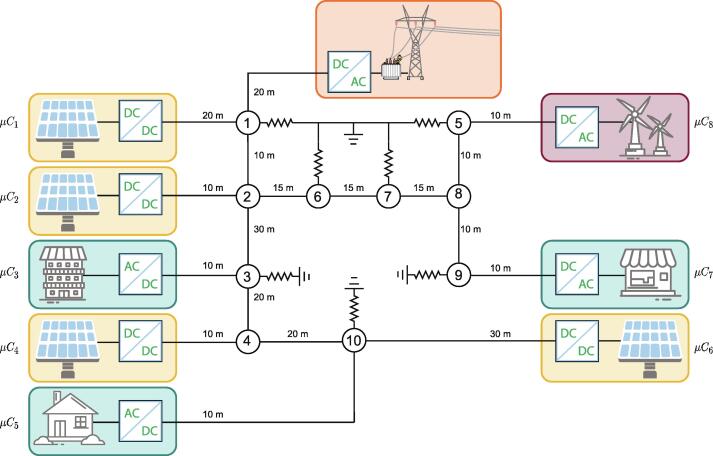
Microgrid with 5 distributed generations units (4 solar and 1 wind), 3 constant power loads and 7 linear loads. Every distributed generations units and constant power loads have a power converter so they can be controlled. The proposed work uses microcontrollers to create communications between the agents while the microgrid distribution, loads and power converters are implemented in a RT-Box 1 from Plecs.

The microgrid system observed in Fig. 1 is the best approach to integrating renewable energies due to their high efficiency, low cost, and the capability to store energy locally and operate on-grid and off-grid. It is a decentralized grid with loads and distributed generation units (DGU) that can be called agents. The DGUs can be controlled, ensuring robustness and stability of the whole microgrid. The agents have a primary control layer whose job is to settle the voltage at a stable point in the system without communicating with other agents. It can also be implemented in higher control layers: the secondary and tertiary layers. This hierarchical control can communicate all the agents to work together to fulfill the microgrid objectives [5]. Since the tertiary control layer also needs the lower control layer, the study will focus on the secondary control layer.

According to its communication protocol, secondary control can be subdivided into three topologies: centralized, distributed, and decentralized secondary control. All these topologies are studied in more detail in [6]. To summarize, the centralized control has a central controller in charge of receiving and processing all the agents’ information and then sending back the control signal to achieve the grid goal. The distributed control eliminates the central controller and communicates the agents with each other; thus, every agent receives and processes the information from others and generates its control signal. Finally, the decentralized control does not have communication. Instead, it uses estimations to predict other agents’ behavior and calculate its control signal.

**Fig. 2:**
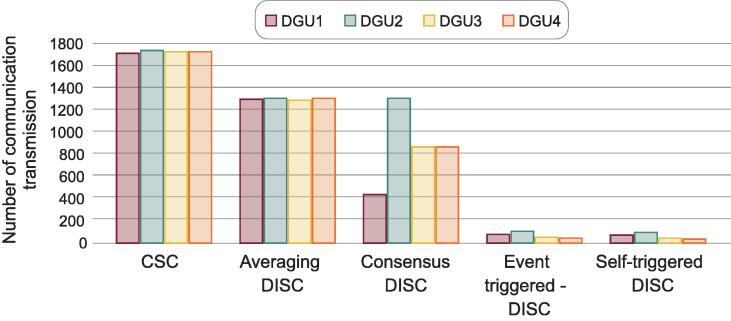
Communication transmission of a microgrid with 4 DGUs under a 150 Hz bandwith periodic communication for centralized secondary controller and 4 distributed topologies, average, consensus, event-triggered and self-triggered [6].

As can be seen in Fig. 2, the centralized topology has the most packet transmissions. By having communication, the centralized and distributed topology have inherent effects such as delays, packet losses, buffer overflow, etc. [7]. Some works try to mitigate these effects by using novel control strategies [8]. However, these effects can destabilize the system and cause a major failure in the microgrid. Nowadays, to analyze stability in systems with communications like microgrids, commonly, the effects previously mentioned are simulated, and there are no protocols like WiFi or Bluetooth implemented.

In recent literature, the work presented in [9] uses hardware-in-the-loop (HIL) and Digital Signal Processors (DSP) to simulate the communications’ inherent effects. Still, they don’t implement real protocols like the ESP-Now from Espressif Systems. On the other hand, in [10], the authors used software-in-the-loop (SIL), where communications are simulated in real-time on one platform and the power electronics on another platform. Then again, there are no real communications protocols used. Several works present remote monitoring systems using open-source platforms for the automation of microgrids [11], [12], [13], [14]. This type of system allows measuring all the relevant variables. Nevertheless, the platform is used to monitor the different variables of the renewable system, but is not used to study the control and stability of the microgrid systems.

Therefore, making a communication dedicated card can help analyze the effects of a communication protocol or faults in the microgrid stability, which can help find the best protocol for a specific situation. In this document, a communication dedicated card is proposed. This control card can implement different communications protocols without modifying the circuit layout. With the use of hardware-in-the-loop, the various supported protocols can be used in complex systems like microgrids, where communication has a considerable effect on the system stability.

The main novelty of our card is that there is currently no single card that integrates all the advantages present in the proposed card, neither in research consulted nor in commercial products. The closest system to the one proposed is the HIL uGrid DSP Interface from Typhoon HIL. This card was specially built for the C2000 family of Texas Instruments (TI) DSP DIM100 cards. The manufacturer suggested working together with the Typhoon HIL emulators to study hierarchical control strategies, communication protocols, and grid standards. Among the supported TI control cards are listed: TMDSCNCD2808 (61 USD), TMDSCNCD28027 (49 USD), TMDSCNCD28035 (80 USD), TMDSCNCD28069 (59 USD), and TMDSCNCD28335 (83 USD). The most economical control card (3 units of TMDSCNCD28027) will be selected to make a fair comparison between the proposed RT Box card and the HIL uGrid DSP Interface from Typhoon HIL, as shown in the following table:

**Table:** 

Features	Proposed RT Box card	HIL uGrid DSP Interface	

Microgrid system			
Maximum number of converters	8	3	
Allows to study communication delays	✓	×	
Allows to study packet losses	✓	×	
Communication protocols between microcontrollers			
Bluetooth	✓	×	
ESP-NOW	✓	×	
CAN BUS	✓	×	
I2C	✓	×	
Possibility of including new protocols	✓	×	
Microcontroller	ESP32 devkit v1	TMDSCNCD28027	
Cores	Dual	Single	
Frequency (MHz)	240	60	
Flash memory (KB)	4000	64	
RAM (KB)	512	12	
ADC resolution	12-bit	12-bit	
Cost per unit (USD)	9	49	
Total price (USD)	300	4100	

## 2 Hardware description

The built-in card is intended to work in conjunction with the RT Box, where the latter has the ability, among other things, to simulate a DC microgrid in real-time with all its parameters and variables. Being able to interact with external elements through the analog and digital ports, so that through these, the interconnection with the built control card is carried out, being able to receive the signals of the voltage and current readings from the RT Box, and send the digital PWM control signals. The joint operation of both platforms makes it possible to implement a wide variety of systems in a simulated environment but outsourcing the control and communication elements to dedicated hardware. On the card, we can also find a Raspberry PI, a cost-effective single board, open-source ecosystem, and well-known computer. Among its features, this computer has two outputs ports:

1. HDMI port helps us make a user graphic interface to visualize the microgrid signals in an external HD monitor in real-time without requiring and additional computer.
2. RJ45 port allows us to connect a computer to monitor the microgrid signals, allowing storage of that data for future analysis. It should be noted that this data can also be saved on an SD card.

**Fig. 3:**
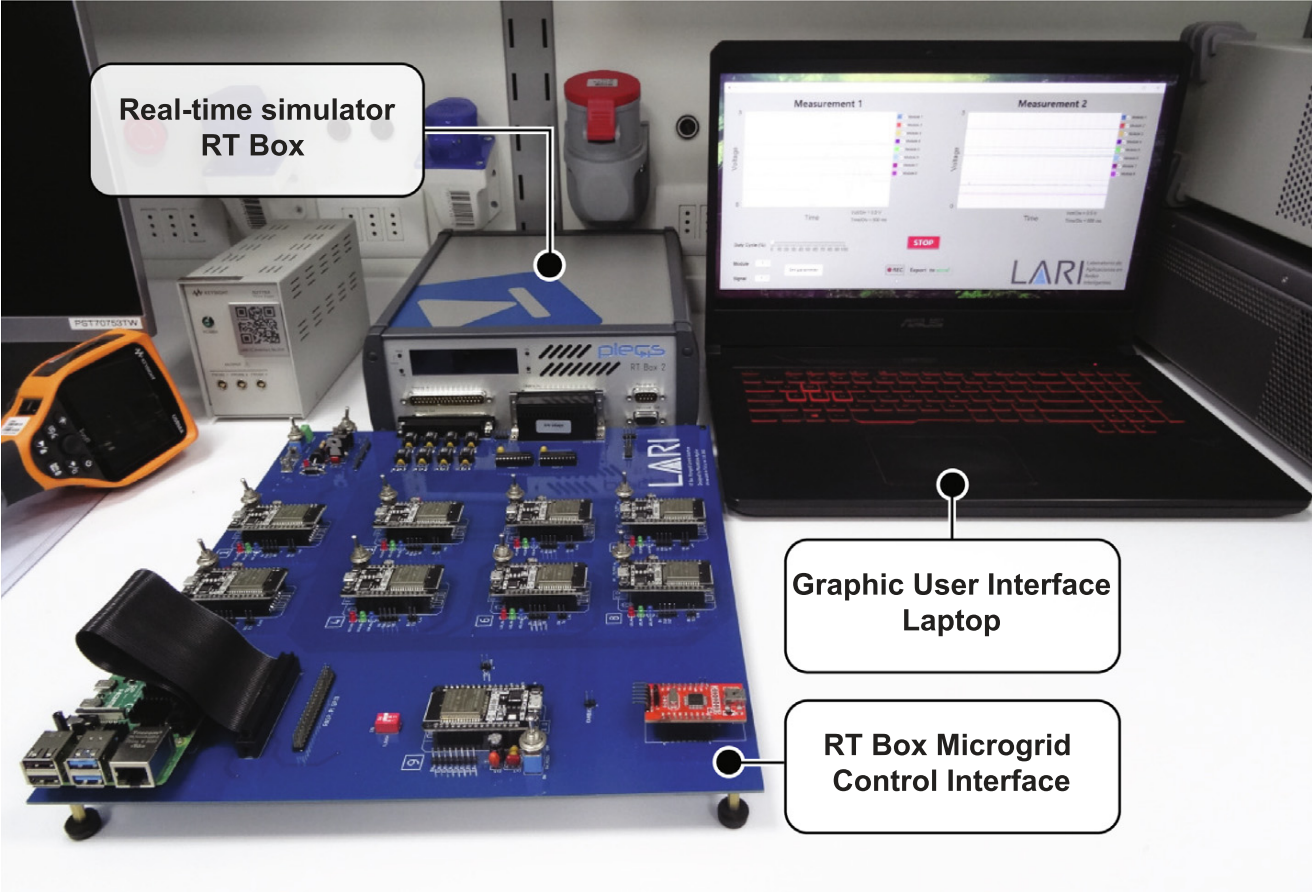
Representation of the total set of elements that make up the interface.

These features make us choose the Raspberry PI as the central controller when implementing a centralized topology because it can work as a central controller and graphical user interface simultaneously. The graphical user interface is implemented using App Designer in MATLAB. Fig. 3 shows the elements mentioned above that participate in the applications destined for the RT Box Microgrid Control Interface.

**Fig. 4:**
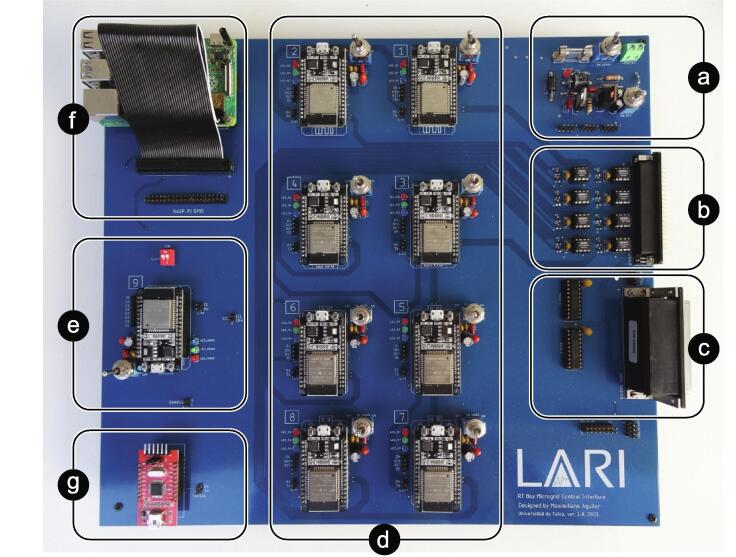
Board top view. (a) Power circuit. (b) Analog input circuit. (c) Digital signals circuit. (d) Control and communication ESP32 micro-controllers. (e) ESP32 communication intermediary micro-controller. (f) Raspberry PI 4 B. (g) USB/ RS232 converter.

The PCB has a design in which its elements can be identified in a modular way. A top view with such identification is given in Fig. 4.

**Fig. 5:**
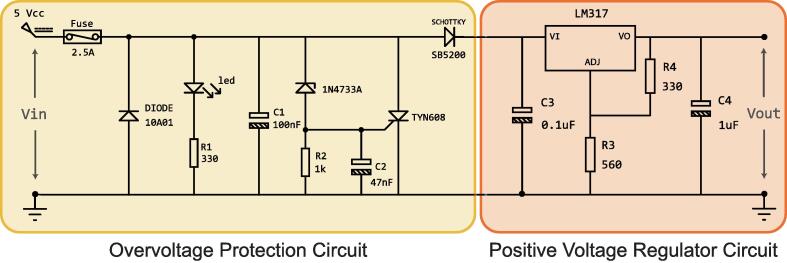
Power circuit. It consists of an over-voltage protection circuit and a 3.3 V voltage regulator circuit.

The printed circuit board (PCB) was designed to work with a power supply of 5 VCC (Fig. 5). Therefore, to ensure the integrity of its components, a circuit protection to suppress supply voltages higher than 5 VCC, plus some margin, was implemented. The margin was established at 0.7 V when considering a Crowbar circuit as an anti-surge system. The components of the PCB, either OPAM’s, ESP32’s transceivers or microcontrollers, work at 3.3 Volt. So the input voltage was regulated at this level, and all devices, except the ESP32, were powered. The power supply circuit is designed to operate in conjunction with the power provided by a laboratory source, since it is complemented with the Source-specific systems for PCB protection. The power circuit has a 10 A rectifier diode inversely connected between the power terminals. If an event causes a short circuit in the power supply, it is connected, reversing the polarities on the PCB power supply, thus preventing damage to PCB components by activating the laboratory source protection systems.

**Fig. 6:**
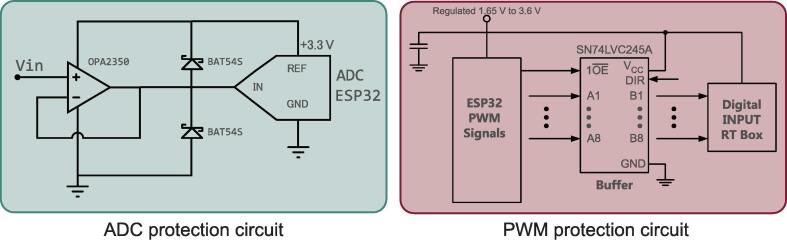
ADC protection circuit of ESP32 micro-controllers, analog inputs.

The ESP32 micro-controllers measure analog voltage and current signals from the RT Box 1, so a protection circuit is necessary against signals that imply an over-voltage. Considering that the ESP32 micro-controllers work in CMOS technology, the input voltages must be at most 3.6 V so as not to damage the ADC (Analog to Digital Converter) of the ESP32. OP2350 Opamps in buffer configuration and BAT54S schottky diodes were used for this work. The implemented circuit is found in Fig. 6 where VIN is the analog signal acquired from the RT BOX. Considering that this circuit protects the input of an analog signal, two same circuits are implemented for each micro-controller since each of the ESP32 will manage two signals.

To improve the quality of the PWM signals generated from the micro-controllers, there are two SN74LVC245A transceivers that act as buffers, each with eight digital channels. When used as a trace termination, these elements allow the decoupling of signals between the micro-controllers and the RT Box 1. In this way, noise and other effects that disturb the signals in their generation and transport are reduced. Furthermore, it is connected to the 16 digital inputs of the RT Box 1. The implemented circuit can be seen in Fig. 6.

**Fig. 7:**
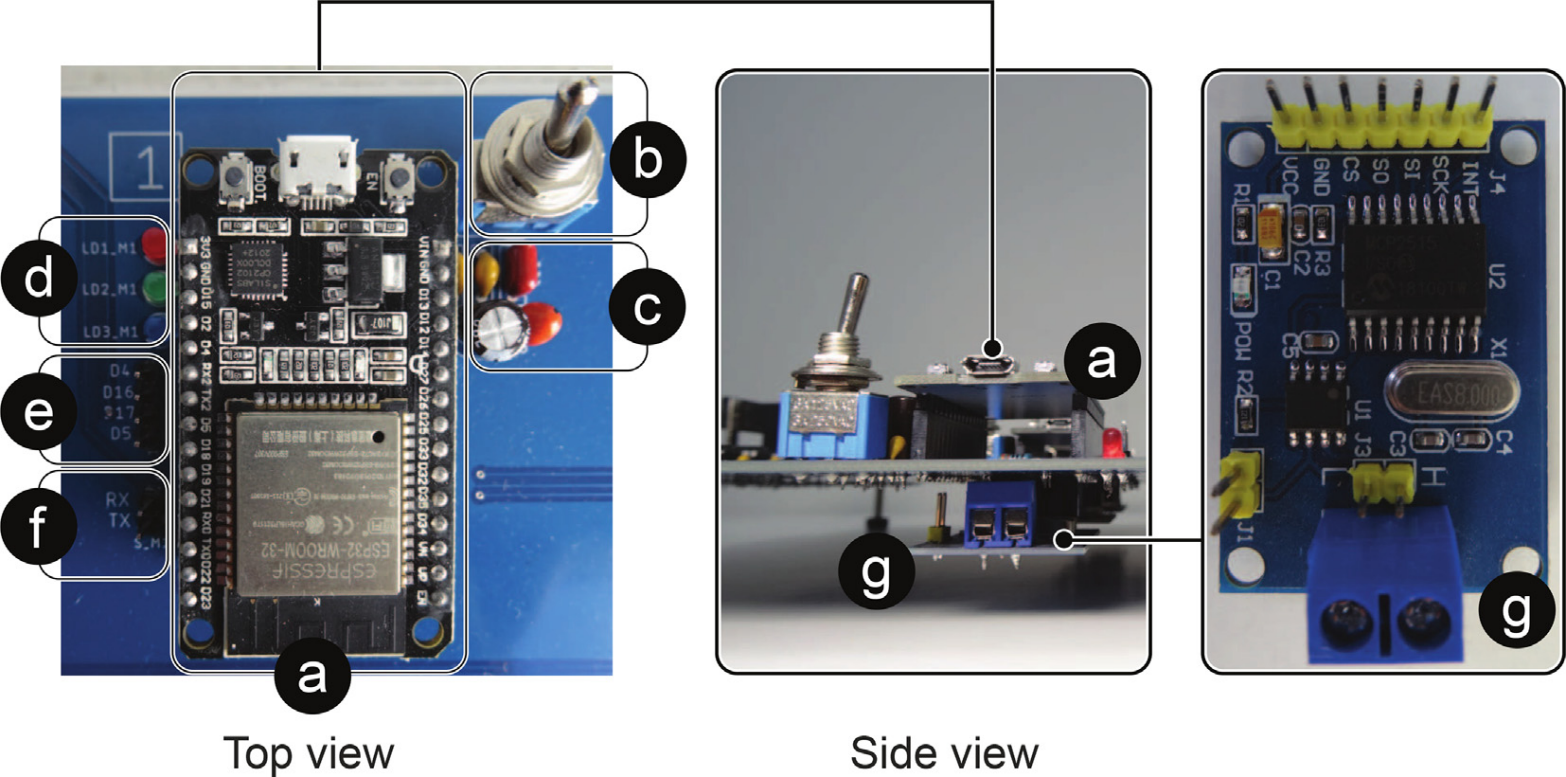
Control and communication micro-controller with its complementary elements. (a) EPS32 Devkit V1 Micro-controller, (b) Micro-controller power switch, (c) Decoupling capacitors, (d) Programmable LEDs from the micro-controller, (e) Available GPIO pins, programmable from micro-controller, (f) Micro-controller UART_0 pins, available to load programs to the micro-controller, (g) MCP2515 module for CAN Bus communication.

The eight control and communication micro-controllers are made up of EPS32 Devkit V1 incorporated into the PCB using removable bases. In addition, all of them have the same series of elements that allow a better interaction in the performance of tests according to the implementations (Fig. 7 - Top View). In Fig. 7 - Side view, it is also possible to differentiate an MCP2515 module for CAN communication, which is located below each of the nine ESP32 micro-controllers included on the PCB.

**Fig. 8:**
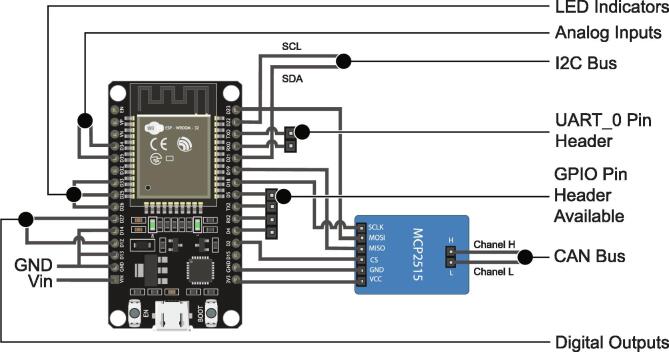
ESP32 standard connection diagram for control and communication.

By means of Fig. 8, it is possible to identify how the control and communication micro-controllers are connected to the different elements of the PCB, such as indicator LEDs, I2C, CAN, UART communication channels, available GPIO pins, digital and analog signals.

A ninth ESP32 microcontroller is used as a communication intermediary between the control microcontrollers and the Raspberry PI 4, this being the only one hundred percent compatible UART (point-to-point) communication between both embedded systems.

**Fig. 9:**
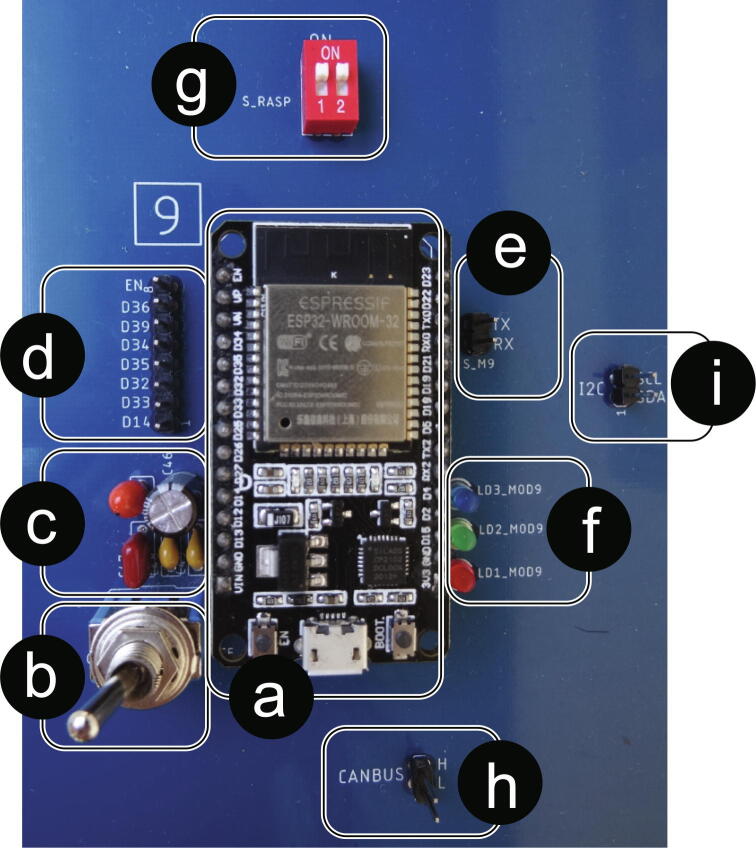
Top view 9th communication intermediary ESP32 micro-controller: (a) EPS32 Devkit V1 Micro-controller, (b) Micro-controller power switch, (c) Decoupling capacitors, (d) Available GPIO pins, programmable from micro-controller, (e) Micro-controller UART _0 pins, available for uploading programs to the micro-controller, (f) Programmable LEDs from the micro-controller, (g) ESP32-Raspberry PI UART Switch, (h) CAN Bus direct access, (i) I2C Bus direct access.

In Fig. 9 this microcontroller can be visualized identifying the elements that accompany it in its implementation on the card.

**Fig. 10:**
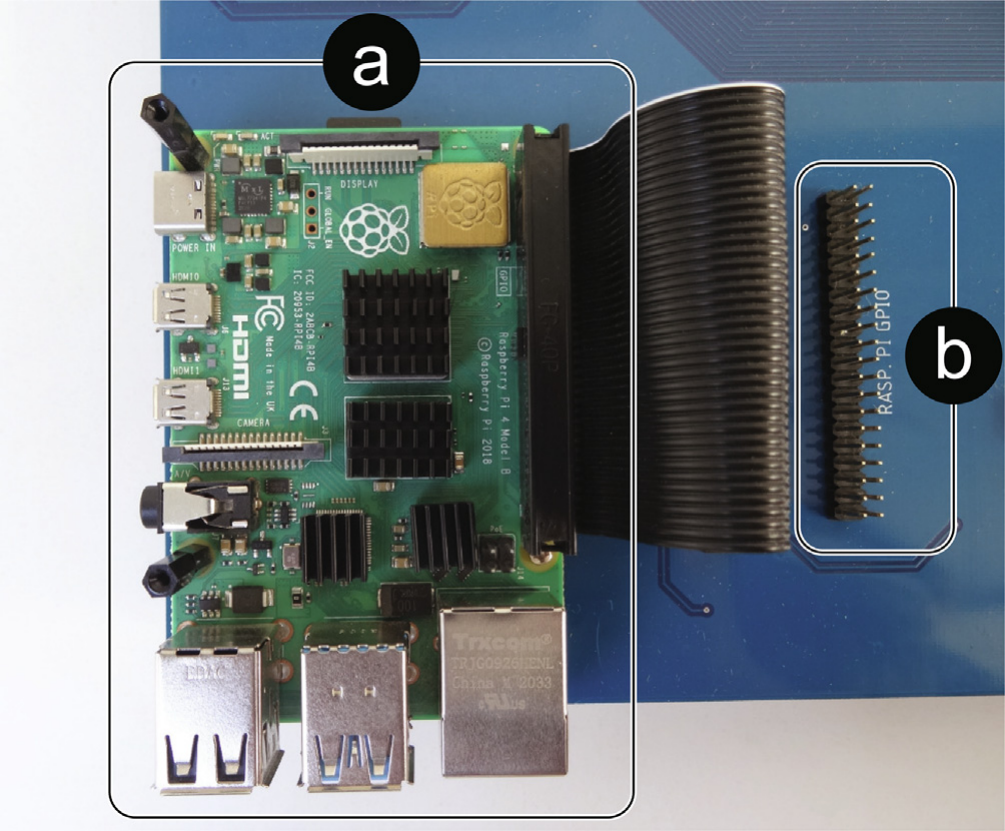
Raspberry PI top view: (a) Raspberry PI 4, (b) Raspberry PI GPIO Pin Header.

**Fig. 11:**
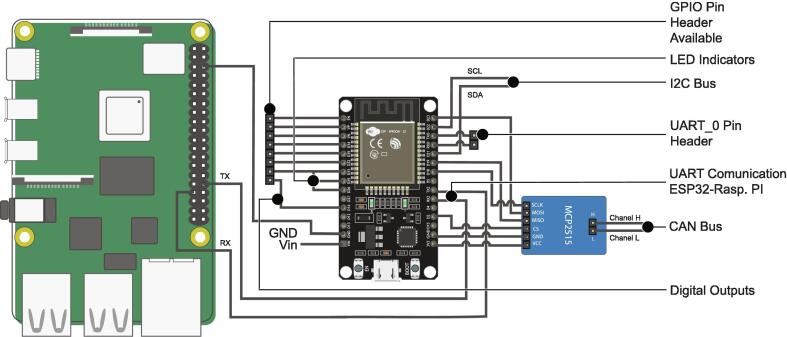
Connection diagram ESP32 communication intermediary and Raspberry Pi connection.

The Raspberry PI is included as an element dedicated to generating a graphical user interface or also to be involved in some micro-grid control task that requires a large computing capacity and/or a centralized scheme (Fig. 10). Fig. 11 shows the existing UART channel connection between the intermediary ESP32 microcontroller and the Raspberry PI, as well as the connections of the intermediary ESP32 with the indicator LEDs, UART communication channels, I2C, CAN Bus and GPIO Pin Header available.

## 3 Design files summary

The design files consist of the documents necessary to build and test the board. It includes the following files:

**Table:** 

Design filename	File type File type	Open Source licence	Location of the file
Schematic.sch	Schematic	CC BY 4.0	https://data.mendeley.com/datasets/mj6mx8d5c5/11
Board.brd	Layout	CC BY 4.0	https://data.mendeley.com/datasets/mj6mx8d5c5/11
Libraries.lbr	Library	CC BY 4.0	https://data.mendeley.com/datasets/mj6mx8d5c5/11
microgrid_gerber.zip	Gerber	CC BY 4.0	https://data.mendeley.com/datasets/mj6mx8d5c5/11
ESP32 file folder	.ino files	CC BY 4.0	https://data.mendeley.com/datasets/mj6mx8d5c5/11
MATLAB file folder	.m files	CC BY 4.0	https://data.mendeley.com/datasets/mj6mx8d5c5/11
Raspberry PI file folder	Python	CC BY 4.0	https://data.mendeley.com/datasets/mj6mx8d5c5/11
PCB explanation and demonstration	MP4	CC BY 4.0	https://data.mendeley.com/datasets/mj6mx8d5c5/11

• Schematic design file, it is Schematic.sch.
• Layout design file, it is Board.brd.
• File with libraries for PCB layout, it is Libraries.lbr.
• Gerber file that contains the necessary information for the manufacture of the printed circuit board, it is microgrid_gerber.zip.
• Programming codes in Arduino IDE of the ESP32 microcontroller, it is ESP32 file folder.
• Programming codes of the graphical interface in MATLAB and codes that make its execution compatible in conjunction with ESP32 microcontrollers, is MATLAB file folder.
• Raspberry PI 4 programming codes, it is Raspberry PI file folder.

## 4 Bill of materials summary

See Table Table 1, Table 2.

**Table 1:** RT Box card bill of materials summary.

Designator	Component	Number	Cost per	Total	Digikey	Material	
			unit	cost	part	type	
				USD	USD	number	
C1, C3, C5, C6, C8, C10, C15, C16, C20, C21, C25, C26, C30, C31, C35, C36, C40, C41, C48, C49, C50, C51, C52, C53, C54, C55, C56, C57, C58, C59	Capacitor, 0.1 μF, 50 V	30	0.47	9.96	399-C3, 18C104, K5R5T, ATR-ND	Ceramic	
C7, C12, C17, C22, C27, C32, C37, C42, C45	Capacitor, 10 μF, 35 V	11	0.55	6.05	478–107, 53–1-ND	Tantalum	
C9, C13, C18, C23, C28, C33, C38, C43, C46	Capacitor, 100 μF, 50 V	9	0.41	3.69	493–125, 70–1-ND	Electrolytic	
C2, C11, C14, C19, C24, C29, C34, C44, C47	Capacitor, 1 μF, 35 V	9	0.71	6.39	EF1105-ND	Polyester	
C4	Capacitor, 1 μF, 50 V	1	0.3	0.3	2368-MLR4, 73K50-ND	Polyester	
R3, R4, R5, R6, R7, R8, R9, R10, R11, R12, R13, R14, R15, R16, R17, R18, R19, R20, R21, R22, R23, R24, R25, R26, R27, R28, R29, R30, R31	Resistor, 330 Ω± 5%, 1/4 W	29	0.02	0.77	CF14JT33, 0RCT-ND	Carbon Film	
R1	Resistor, 330 Ω± 5%, 1/2 W	1	0.1	0.1	CF12JT33, 0RCT-ND	Carbon Film	
R2	Resistor, 560 Ω± 5 % 1/2 W	1	0.1	0.1	S560H, CT-ND	Carbon Film	
-POWER+	TERM, BLOCK 2P, HORIZON, 2.54MM, PCB	1	1.11	1.11	732–69121, 09100, 02-ND

**Table 2:** RT Box card bill of materials summary (continued).

Designator	Component	Number	Cost per	Total	Digikey	Material		
			unit	cost	part	type		
				USD	USD	number		
Z5.1	Zener Diode, 5.1 V, 500 mW	1	0.09	0.09	2156-1N5, 993D-ND			
DIGITAL_PORT	37-pin D-Sub, Stacked	1	10.73	10.73	AE109, 67-ND			
ANALOG_PORT	37-pin D-Sub, Male	1	1.45	1.45	AE109, 88-ND			
D1, D2, D3, D4, D5, D6, D7, D8, D9, D10, D11, D12, D13, D14, D15, D16	DIODE, ARRAY, SCHOTTKY 30 V	16	0.22	0.22	2156-BAT, 54S-ND			
BOARD1, BOARD2, BOARD3, BOARD4, BOARD5, BOARD6, BOARD7, BOARD8, BOARD9	ESP32, WROOM, DEVKIT, V1 30 pin	9	10	90				
FUSE	Fuse glass, 2.5 A	1	1.76	1.76	283–50, 72-ND			
FT232BL	RS232-USB	1	5.90	5.90	FT232BL			
	MCP2515 Can Bus Interface Module	9	5.95	53.55				
	Raspberry pi 4B - 8gb	1	75.00	75.00				

## 5 Build instructions

### 5.1 Original design construction

To carry out the construction of the card, considering the original design without modifications, it is only necessary to provide an electronic card manufacturer with the microgrid_gerber.zip file. However, suppose you also want the manufacturer to be in charge of the acquisition and assembly of components. In that case, you must provide the list of components in the “ Bill of materials summary ” section.

#### 5.1.1 Component assembly

**Fig. 12:**
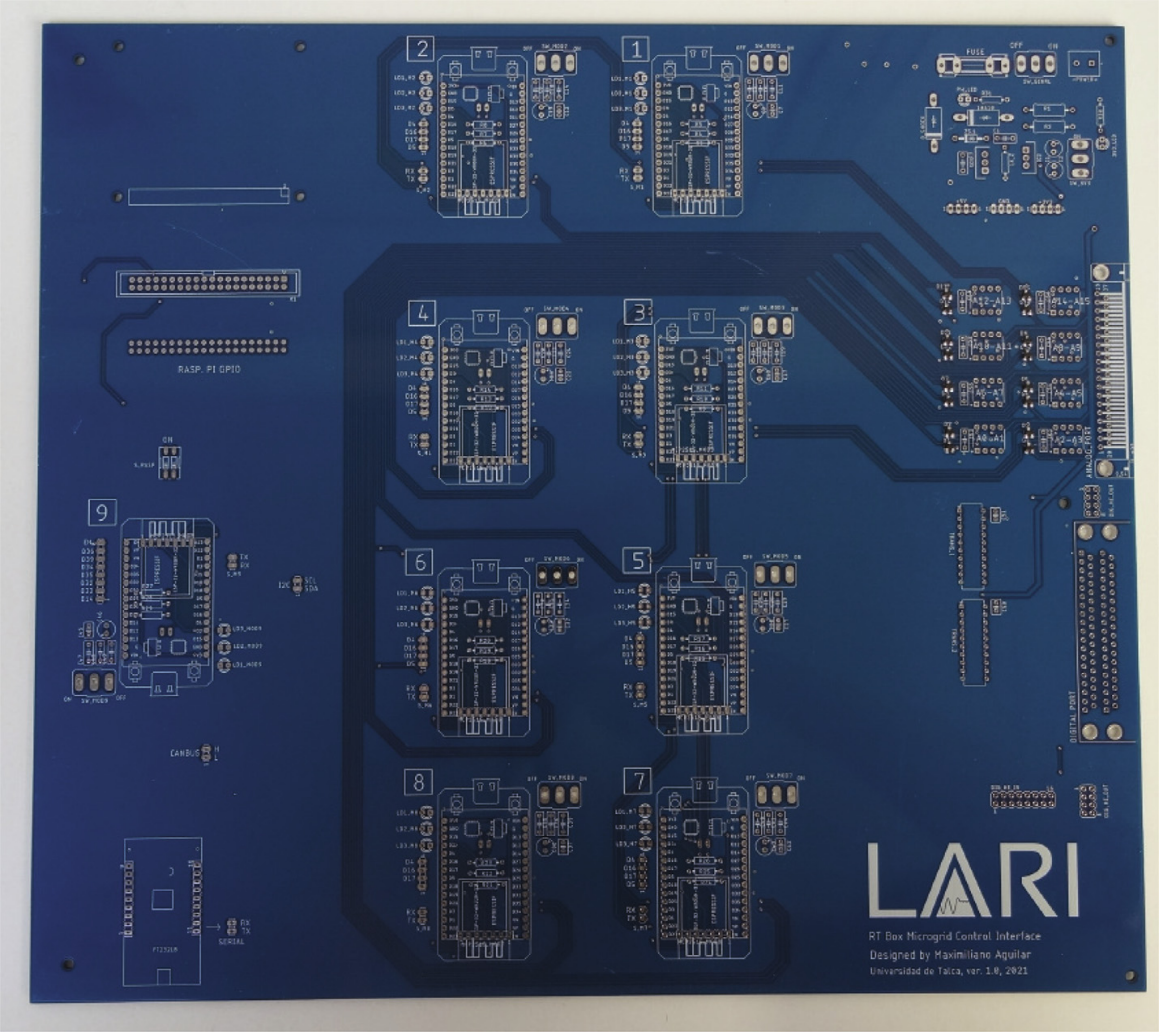
Electronic card without components.

If you choose to purchase and assemble the components yourself upon receipt of the card, it will have an identical appearance to that of Fig. 12, and the color of the chosen anti-solder mask may vary.

The assembly of the components can be done guided by the silkscreen printing with the silhouette of the components on the card, these being located on the TOP layer and welded on the BOTTOM layer as they are THT (Through-Hole Technology) components, with the exception of the BAT54S, which are smd components, so they are welded on the TOP layer.

**Fig. 13:**
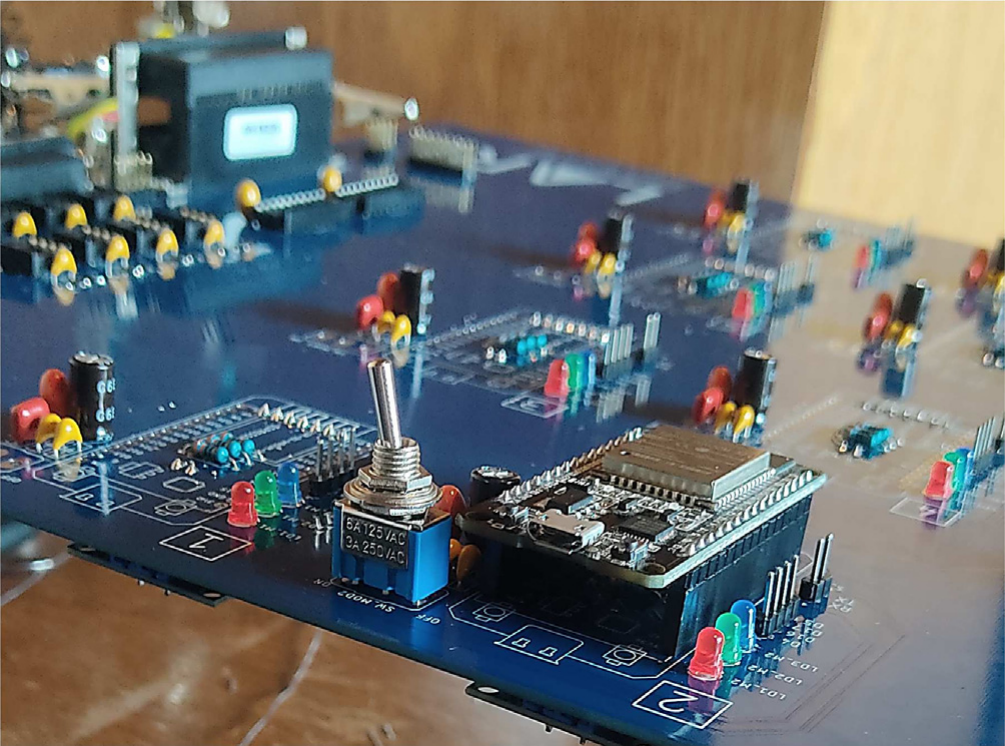
Electronic card in soldering process, top view.

There are components such as the OPA2350PA, SN74LVC245AN, FT232LB, MCP2515 and ESP32’s that are not soldered directly on the board. Still, some bases are soldered in their place, bases on which these components are then connected. In this way, these components can be disassembled and easily replaced from the card. A case of the above can be seen in Fig. 13, where the ESP32 micro-controllers are mounted on a female pin header base.

**Fig. 14:**
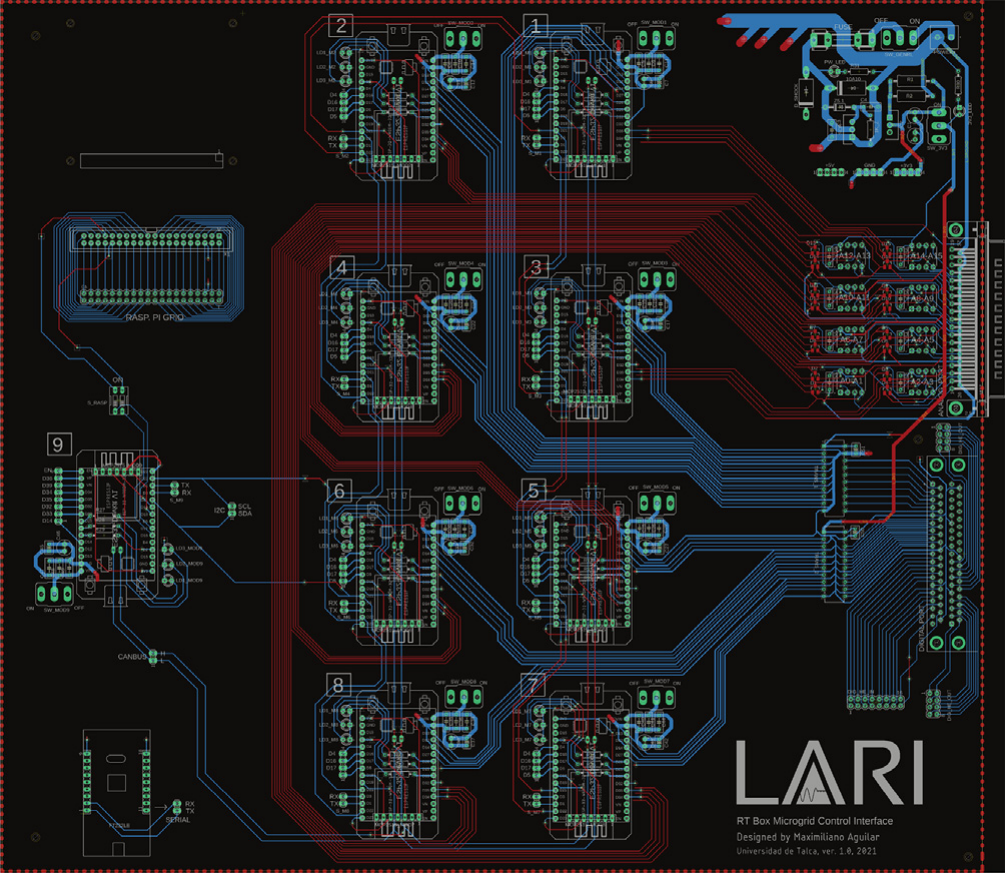
Design view in Eagle software.

To guide yourself in the welding process, it is useful to review the microgrid.brd file with Eagle 9 or higher, as shown in Fig. 14.

#### 5.1.2 Raspberry PI assembly

The Raspberry PI is attached to the card using hexagonal standoffs and connected via a flexible 40-way connector to the available port on the card.

### 5.2 Construction modifying the design

If you want to modify the design, you need to have the files microgrid.sch, microgrid.brd and libraries.lbr in the same folder. From Eagle 9.6 (or higher) the microgrid.sch file should be opened and in the upper panel follow the paths:

Library→Openlibrarymanager→Browse

Locate the file libraries.lbr and select the ” USE ” button. In this way the library with the components used will be loaded.

Additionally, when making modifications to the schematic design, when pressing the Generate/switch to board button, the changes will be reflected in the existing microgrid.brd file.

#### 5.2.1 Gerber file generation

**Fig. 15:**
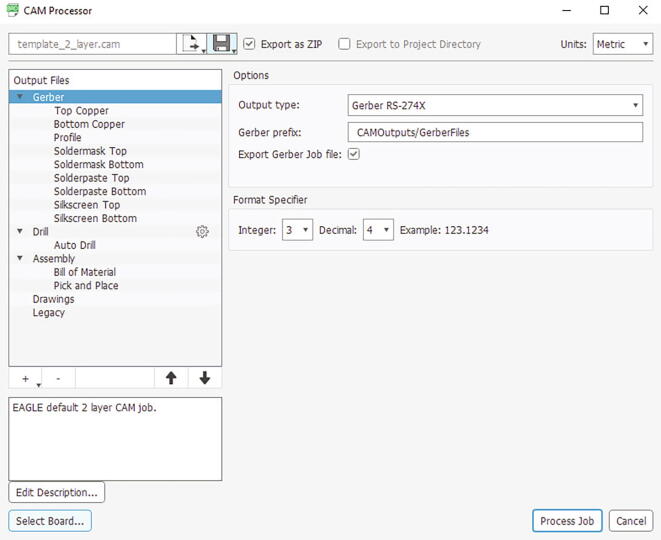
CAM Processor panel view.

Once the modifications to the schematic design and layout design have been made, the Gerber file must be generated, which will be the set of files to be delivered to the electronic card manufacturer. This file is generated by pressing the ” CAM Processor ” button from the Board screen in Eagle and introducing the parameters as shown in Fig. 15, then pressing the ” Process Job ” button will request the path where the file will be saved and when selected, the program will generate the set of Gerber files compressed in a.zip file in the chosen path.

The rest of the actions must be carried out according to the instructions for the construction of the original design.

## 6 Operation instructions

The following are crucial elements for the card’s operation, which can be specifically complemented with the video “ Introduction to RT Box Microgrid Control Interface ”, which can be extracted from the set of videos categorized within the PCB filename explanation and demonstration.

### 6.1 Energy supply for the card

Powering the card through a laboratory source is recommended to have the latter’s protection elements against short circuits or overvoltages. The power supply must supply 5 VDC/ 2.5 A.

The card must be fed through the terminal block identified in Fig. 5. Then, the positive and negative terminals are identified in the silkscreen that accompanies it. In the event of a reverse power supply, the card has a series fuse and a reverse polarized diode in parallel, so a short circuit will be generated, where the laboratory source will cut off the power supply as a protection measure or the fuse it will melt and open the circuit.

On one side of the terminal block there is a switch identified as SW_GENRL, which enables or disables the passage of current to all the components of the card, except for the Raspberry PI, so as a protection measure, the switch must be disabled when connecting the cables from the power supply to the terminal block. Once the connection has been made correctly, the red indicator LED should light up when the switch is activated, called PW_LED in the silkscreen. The card has a 3.3 V voltage regulator circuit, a voltage that some components need to operate. Being possible to enable or disable the power supply of these components employing the SW_3V3 switch, having the green indicative led 3V3_LED.

In addition, each ESP32 micro-controller has its own power switch, with the possibility of independently disabling each micro-controller.

### 6.2 Energy supply for Raspberry PI

The Raspberry PI requires independent power through a transformer that delivers 5 VDC/3 A, which must be connected to the Raspberry PI 4 through its USB-C port. Therefore, it is recommended to use the official transformer.

### 6.3 ESP32 programming environment

The micro-controllers of the ESP32 line have official software for their programming, provided by Espressif called ESP-IDF (Espressif IoT Development Framework), which is available for Windows, Linux, and Mac OS. On the other hand, the popular and cross-platform Arduino IDE software also has official support for programming this micro-controller line.

Although ESP-IDF has more debugging tools and FreeRTOS for professional IoT projects, for this project, the Arduino IDE has been chosen, due to affinity, comfort and experience in the software, as well as its large amount of documentation, community, support for any implementation that is required and that Espressif has integrated into this software the ability to work with the second core of ESP32.

#### 6.3.1 Installing ESP32 on Arduino IDE

Assuming that you have the latest version of the software, the card is included in the following section of the Arduino IDE:

File→Preferences→AdditionalCardURLsManager

**Fig. 16:**
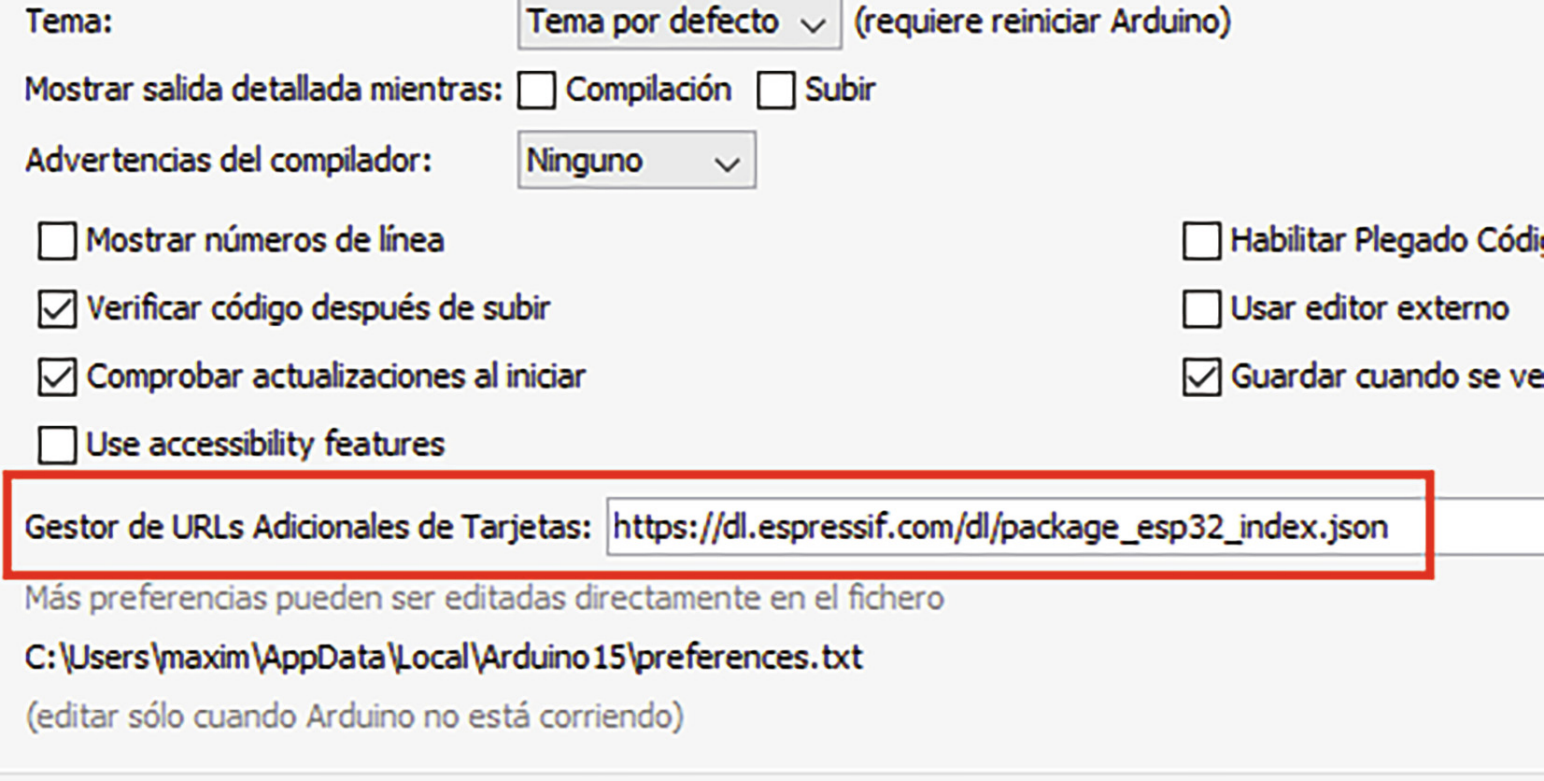
Including the ESP32 file in Arduino IDE.

- As shown in Fig. 16, entering the URL in the box:

https://dl.espressif.com/dl/package_esp32_index.json

- Then back in the main window of the program, and access:

Tools→Board→BoardManager

**Fig. 17:**
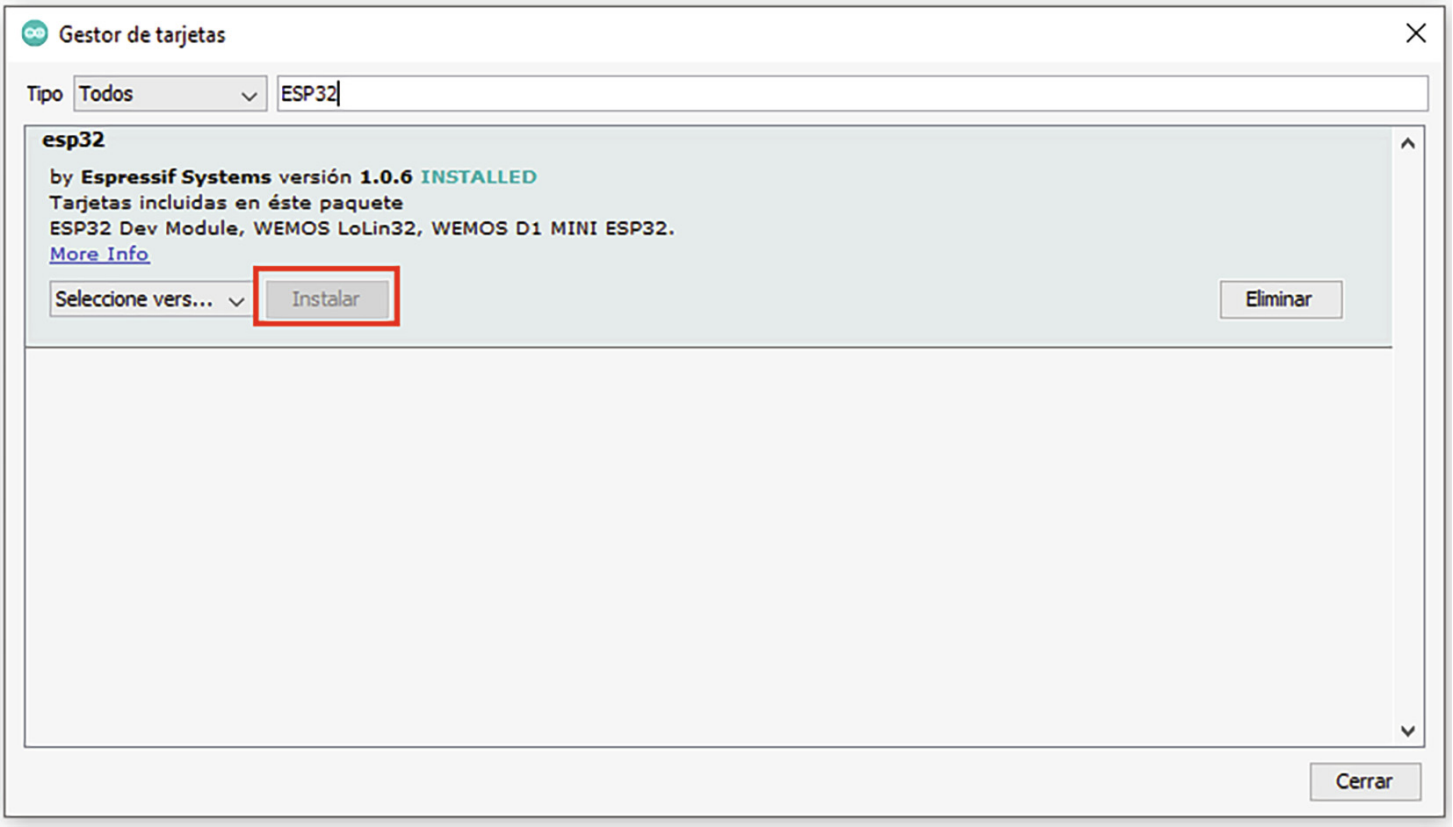
Installing ESP32 on Arduino IDE.

- Entering **ESP32** in the search bar and installing **esp32 by Espressif Systems**, as shown in Fig. 17.

#### 6.3.2 Loading a code for ESP32 in Arduino IDE

**Fig. 18:**
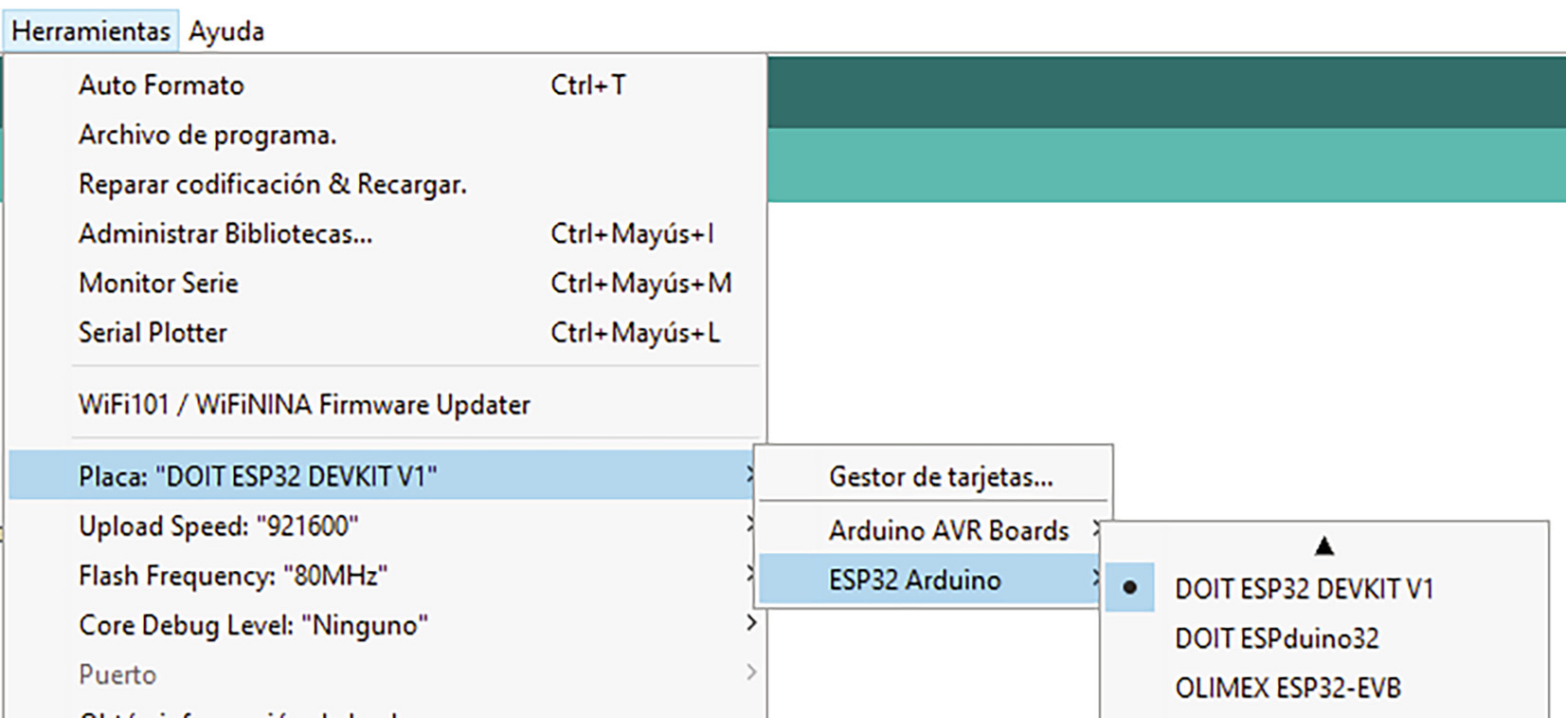
Selecting board model.

Since the ESP32 has many models and versions, both official and third-party, it is necessary to choose the specific model of our micro-controller so that the code can be loaded on the card correctly. In Fig. 18 you can see how to select our model.

**Fig. 19:**
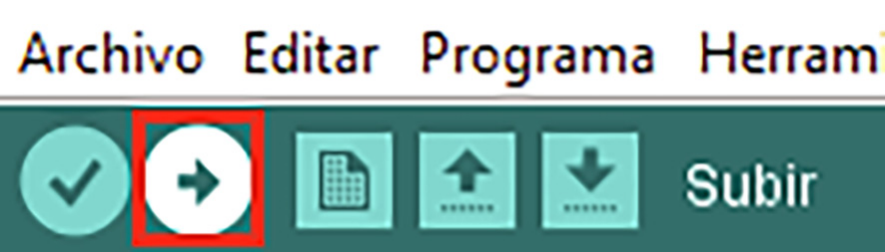
Uploading program.

Once the code is available, the program is loaded onto the card, clicking on Upload (Fig. 19).Upload program via direct USB connection

**Fig. 20:**
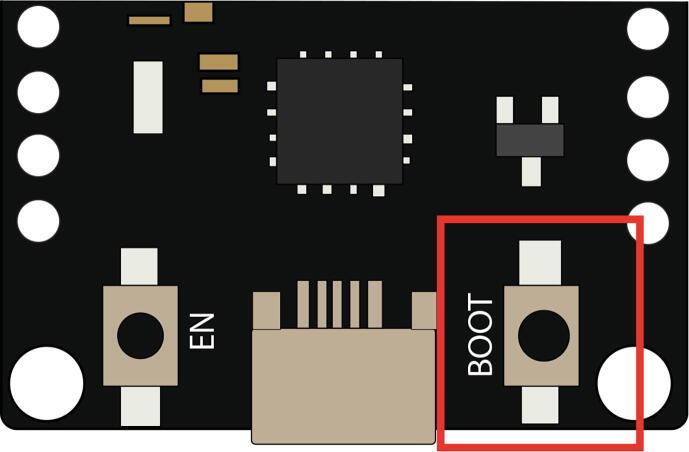
Button to be pressed on the micro-controller to load programs.

An important detail when loading a program into this micro-controller is that while a sketch is being uploaded (not compiled), the BOOT button on the card must be held down (Fig. 20) until the message appears *’Writing at* *direction**’* in the lower section of the software. This situation occurs since this model has problems entering programming mode, so the manufacturer has provided the button option. On the internet, you can find another solution that consists of connecting a 10uF capacitor between the EN and GND pins, but this option did not work in the development of this project.Upload program through USB/RS232 converter

Another way to upload a program is through an adapter that connects directly to the UART _0 pins of the ESP32. This option facilitates the implementation by handling the final PCB with the 9 ESP32 since the USB cable from the computer connects only to this converter, and what is alternated is the connection of these pins through a cable.

To load a program using this method, press the BOOT button, and when the message *’Connecting …’* appears, stop pressing the BOOT button, and press once the EN button.

## 7 Validation and characterization

**Fig. 21:**
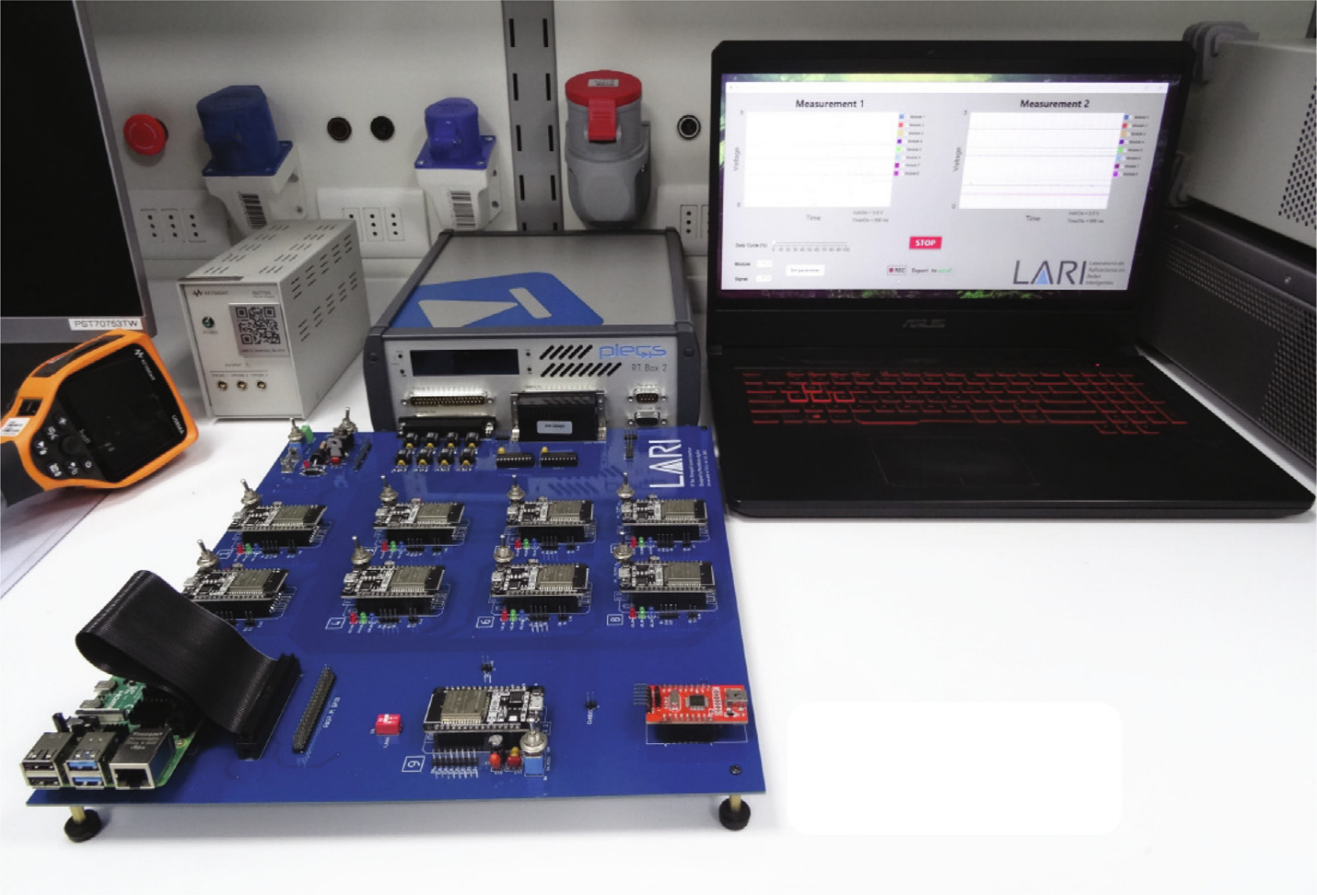
Introduction video to the RT Box microgrid control interface. Link: https://data.mendeley.com/datasets/mj6mx8d5c5/11.
• **I2C - RT Box Microgrid Control Interface**
  This case explains how the I2C protocol has been implemented on the board, its capabilities, limitations, and considerations. In addition to this, the communication between the microcontrollers and Raspberry Pi is demonstrated, in interaction with the graphic interface. All this is seen in the video in Fig. 22 (see link).

• **Introduction to RT Box Microgrid Control Interface**
  In the video in Fig. 21 (see link), the general capabilities of the card are detailed, the sectors that compose it are identified, and the graphic interface developed is also reviewed, explaining its capabilities and tools.

**Fig. 22:**
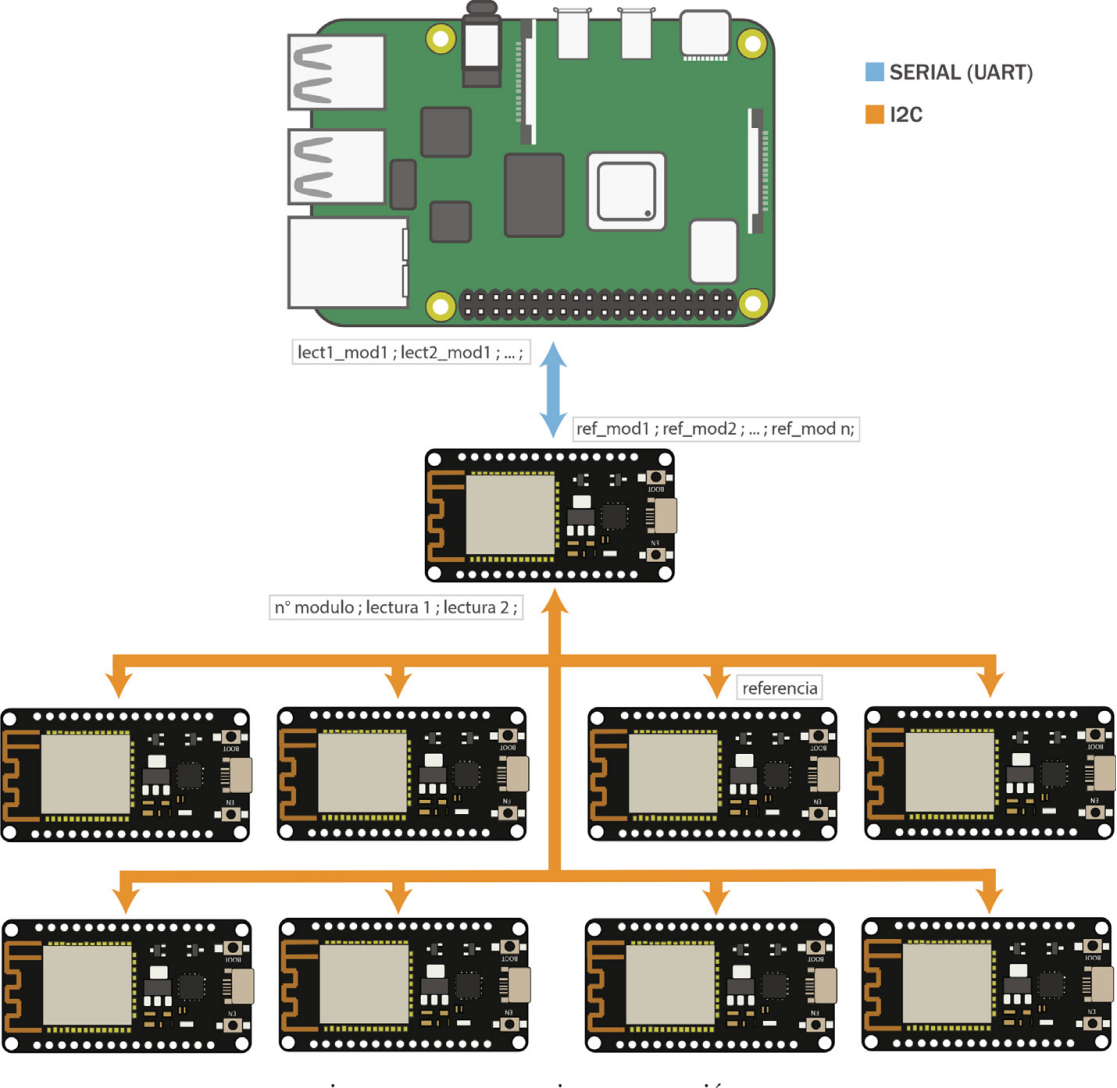
I2C communication protocol operation video. Link: https://data.mendeley.com/datasets/mj6mx8d5c5/11.
• **ESPNOW - RT Box Microgrid Control Interface**
  In the video in Fig. 23 (see link), a review of the ESPNOW protocol operation is made, showing the network configuration that has been implemented together with the graphical interface.

**Fig. 23:**
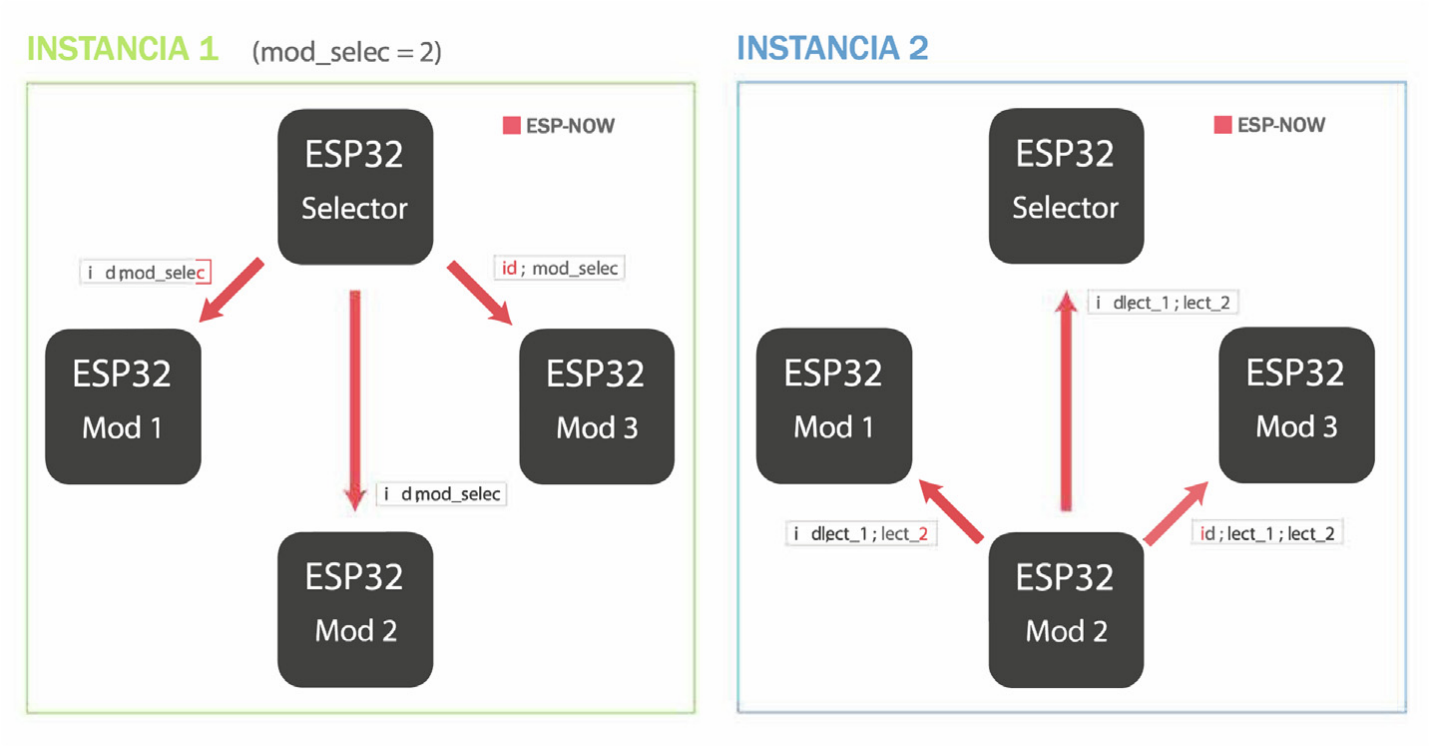
ESPNOW communication protocol operation video. Link: https://data.mendeley.com/datasets/mj6mx8d5c5/11.
• **CAN Bus - RT Box Microgrid Control Interface**
  For the CAN bus communication protocol, it is performed a review of its operation and also the demonstration in conjunction with the graphical interface created. The video in Fig. 24 (see link) allows us to appreciate this and see its main characteristics.

**Fig. 24:**
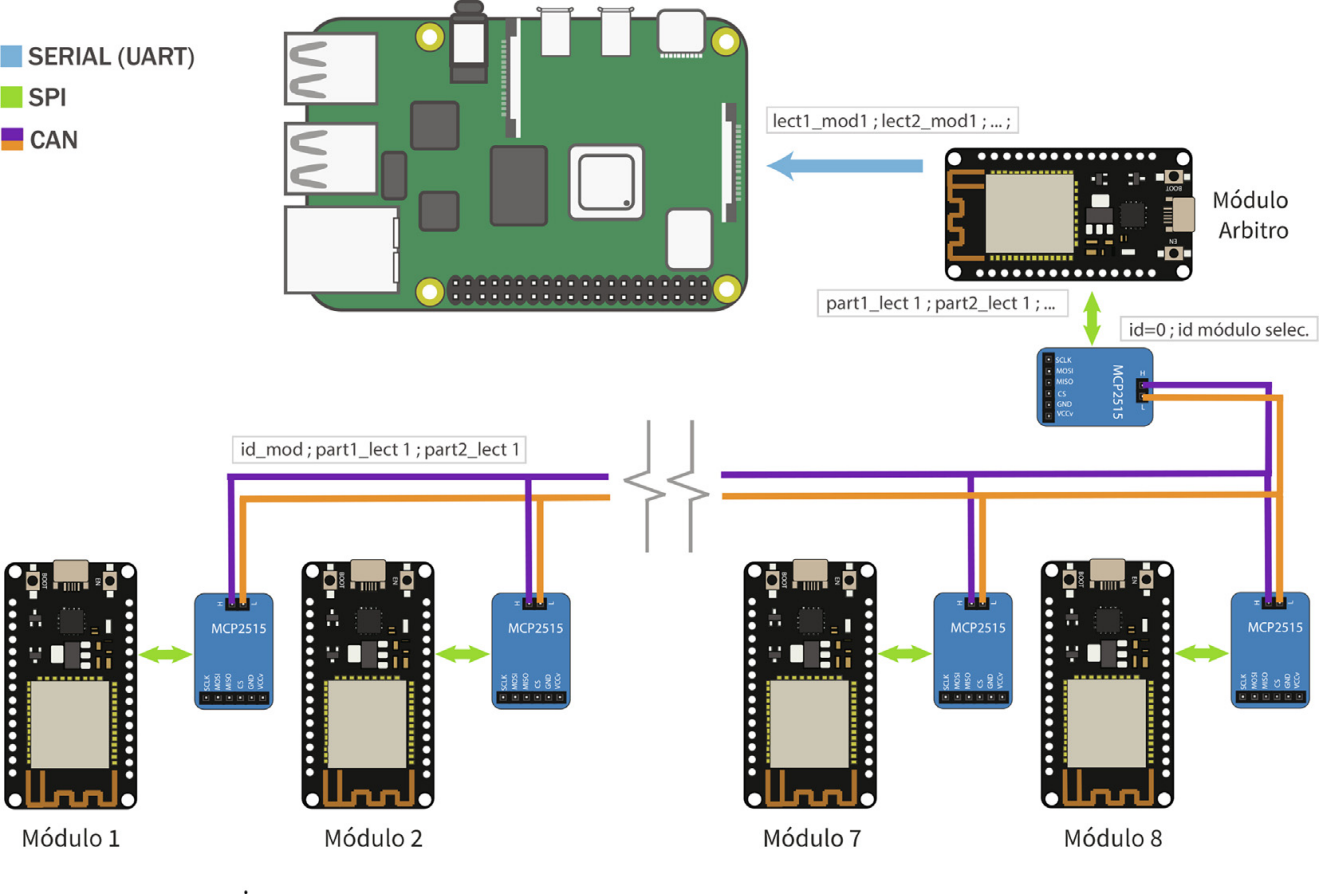
CAN communication protocol operation video. Link: https://data.mendeley.com/datasets/mj6mx8d5c5/11.
• **Bluetooth - RT Box Microgrid Control Interface**

**Fig. 25:**
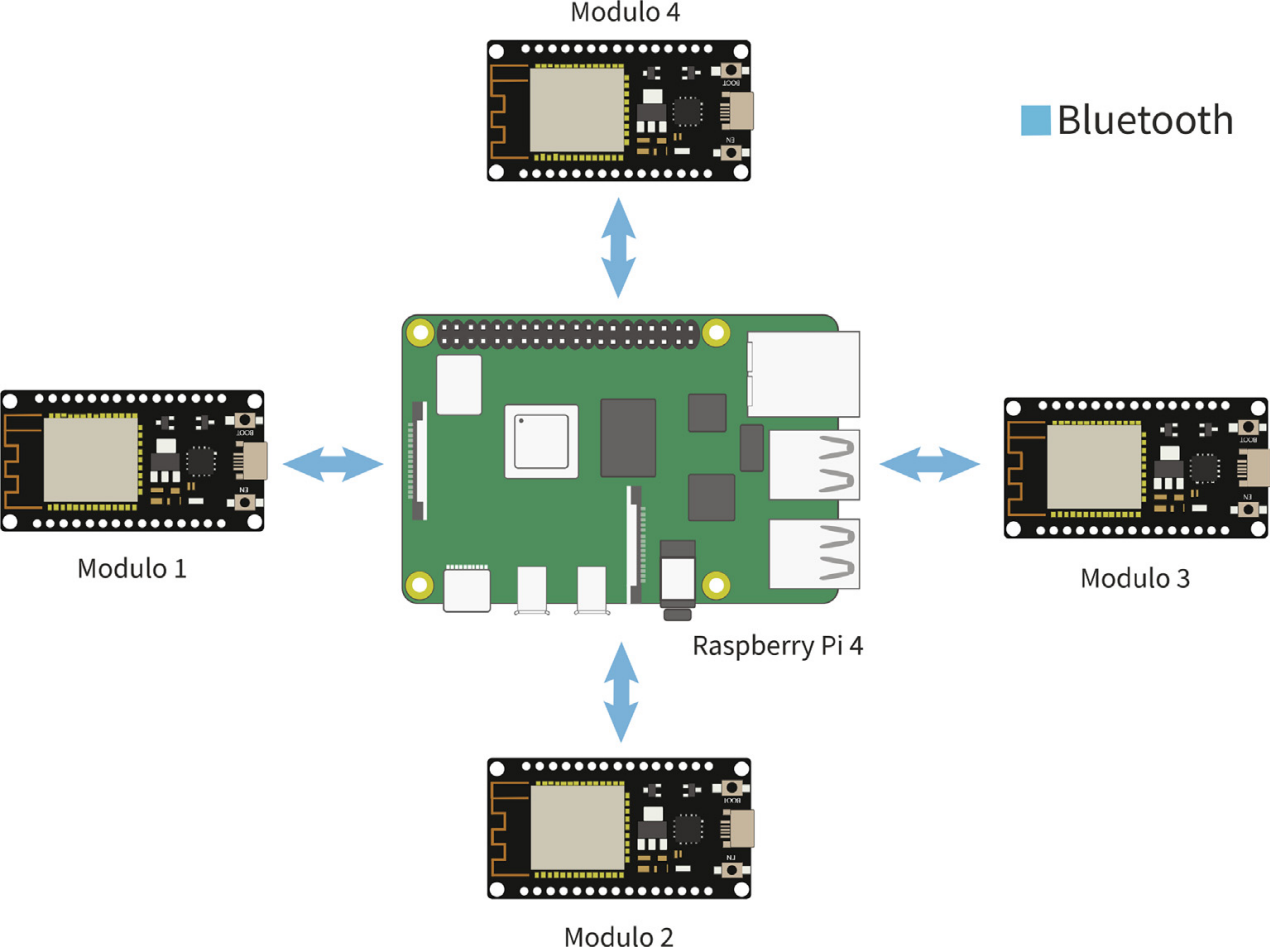
Bluetooth communication protocol operation video. Link: https://data.mendeley.com/datasets/mj6mx8d5c5/11.

The video in Fig. 25 (see link) explains how the Bluetooth communication protocol has been implemented on the board, its capabilities, limitations, and considerations.

## 8 Measurement result and analysis on system performance

**Fig. 26:**
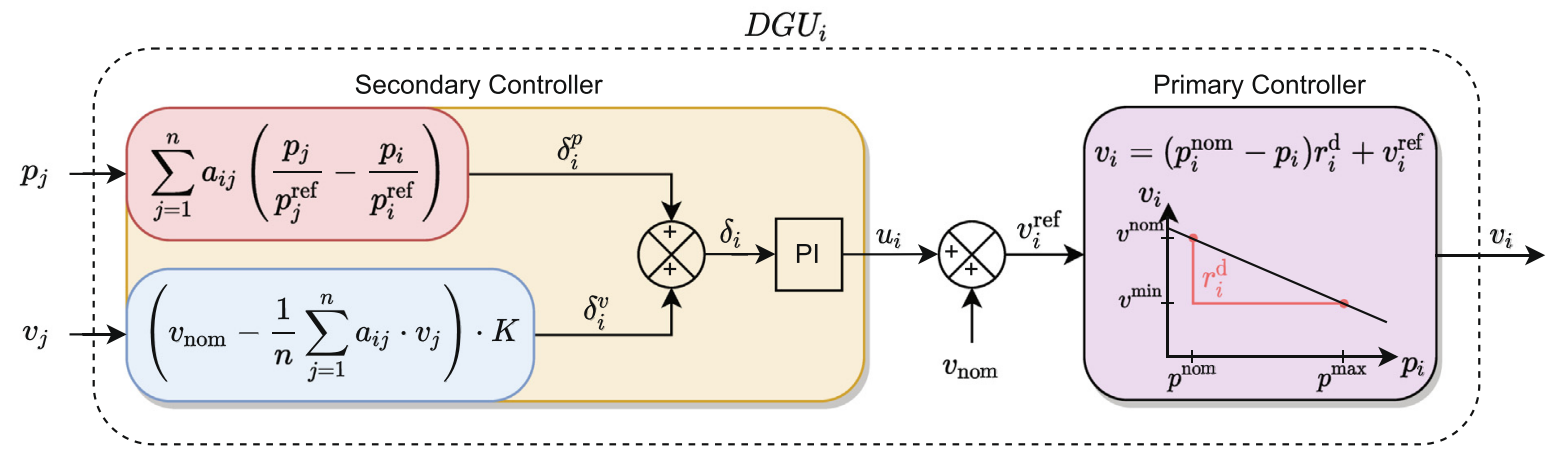
Control law used in the distributed secondary controller based on consensus and average voltage.

**Fig. 27:**
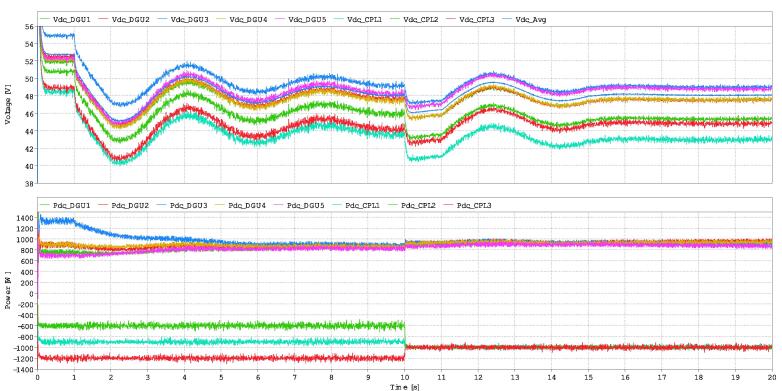
Results with 5 DGUs and 3 constant power loads with a communication delay of 1000 ms. At 1 s the secondary control is activated. At 10 s there is a change in power of the constant power loads. Finally, at second 15, packets losses of 80 % are included.

Some results of the RT-Box microgrid control interface are presented in Fig. 27. The results were developed with the microgrid and eight commutated converters, as is seen in Fig. 1, where converters in nodes 3, 4, and 9 are defined as constant power loads. The results show the voltage and power for each agent when the distributed secondary control layer is activated at 1 s. The control method used to obtain the results is based on consensus with voltage average, presented in Fig. 26. The objective of the control is to maintain a correct power-sharing and dc bus voltage [15]. The communications made are all the agents with all. Each agent can send and receive from any other agent; this increases the system’s stability, speed, and robustness. The results show how delays can affect the system’s response, making it oscillatory and overshot. When the packet losses come in, the system becomes more stable. This is because packets losses get rid of the secondary controller and its delays, leaving most of the time (80 %) the droop control reference only. Nevertheless, the system reaches the average voltage reference and produces correct power-sharing between the DGUs.

Control parameters like PI gains have to be adjusted to obtain stability on the microgrid. Because of the non-linear effect of constant power loads, criteria such as Lyapunov asymptotic stability can be used to ensure stability according to the communications delays [16], [17].

## 9 Future Challenges and Works in Progress

Future challenge work is being able to connect the board to various hardware-in-the-loop simulation tools such as dSpace, OPAL-RT, Speedgoat, and Typhoon HiL, among others. The difficulty of this task is to adapt the card connectors, designed to be compatible with the RT-Box, with different connectors configuration presented in other HIL tools. This task can be addressed by creating cables that make the connector’s interconnection compatible.

Another future work will also improve the connectivity of the RT Box card by including additional communication types (wired and wireless) using new module shields. The new communication protocols should be according to the microgrid control layer architecture [18]. These modules can connect each of the microcontrollers with external connector pins similar to the actual MCP2515 module for the CAN Bus communication.

Although the proposed RT Box card has designed to control a microgrid system, its use can be extended to other power electronics applications. In this context, the energy management of electric vehicles, satellites, data centers, and photovoltaic power modules interconnection, among others, can be studied in future works with the proposed card. Finally, in the context of microgrids, interesting future works such as the research on cybersecurity, the design of new digital converter controls, or the operation under critical conditions such as island mode and failure conditions, among others, can be covered by the proposed RT Box card.

## CRediT authorship contribution statement

**Maximiliano Aguilar:** Conceptualization, Methodology, Software, Validation. **Sebastián Riffo:** Investigation, Conceptualization. **Antonio Veliz:** Writing - original draft, Formal analysis. **Catalina González-Casta**ñ**o:** Writing - original draft, Resources, Funding acquisition. **Carlos Restrepo:** Conceptualization, Methodology, Resources, Supervision.

## Declaration of Competing Interest

The authors declare that they have no known competing financial interests or personal relationships that could have appeared to influence the work reported in this paper.

## References

[1] Jahid A., Monju M.K.H., Hossain M.E., Hossain M.F. (2018). Renewable energy assisted cost aware sustainable off-grid base stations with energy cooperation. IEEE Access.

[2] S. Kouro, J. Leon, D. Vinnikov, and L. Franquelo, ”Grid-connected photovoltaic systems: An overview of recent research and emerging pv converter technology,” IEEE Industrial Electronics Magazine, vol. 9, pp. 47–61, 03 2015.

[3] V. Yaramasu, B. Wu, P.C. Sen, S. Kouro, and M. Narimani, ”High-power wind energy conversion systems: State-of-the-art and emerging technologies,” Proceedings of the IEEE, vol. 103, no. 5, pp. 740–788, 2015.

[4] IRENAS, “Renewable energy statistics 2021.” Available at URL:https://irena.org/publications/2021/Aug/Renewable-energy-statistics-2021 (Ago. 2021).

[5] Meng L., Shafiee Q., Trecate G.F., Karimi H., Fulwani D., Lu X., Guerrero J.M. (2017). Review on control of dc microgrids and multiple microgrid clusters. IEEE Journal of Emerging and Selected Topics in Power Electronics.

[6] Khayat Y., Shafiee Q., Heydari R., Naderi M., Dragičević T., Simpson-Porco J.W., Dörfler F., Fathi M., Blaabjerg F., Guerrero J.M., Bevrani H. (2020). On the secondary control architectures of ac microgrids: An overview. IEEE Transactions on Power Electronics.

[7] Saleh M., Esa Y., Mohamed A. (2018). 2018 IEEE Energy Conversion Congress and Exposition (ECCE).

[8] Duan J., Chow M.-Y. (2020). Robust consensus-based distributed energy management for microgrids with packet losses tolerance. IEEE Transactions on Smart Grid.

[9] Lou G., Gu W., Lu X., Xu Y., Hong H. (2020). Distributed secondary voltage control in islanded microgrids with consideration of communication network and time delays. IEEE Transactions on Smart Grid.

[10] R. Thiagarajan, ”Real-time simulation of a smart inverter,” 2017.

[11] G.C.G. d. Melo, I.C. Torres, Í. B.Q. d. Araújo, D.B. Brito, and E. d. A. Barboza, “A low-cost iot system for real-time monitoring of climatic variables and photovoltaic generation for smart grid application,” Sensors, vol. 21, no. 9, p. 3293, 2021. 10.3390/s21093293 PMC8126226 34068743

[12] Portalo J.M., González I., Calderón A.J. (2021). Monitoring system for tracking a pv generator in an experimental smart microgrid: An open-source solution. Sustainability.

[13] González I., Calderón A.J. (2019). Integration of open source hardware arduino platform in automation systems applied to smart grids/micro-grids. Sustainable Energy Technologies and Assessments.

[14] Luna-Gonzalez M.L., Becerra-Bayona S.M., Serrano-Diaz N., Lobo-Quintero R.A. (2020). Implementación de tecnologías libres y sensores remotos para un biobanco: el desafío de producir a bajo costo. Información tecnológica.

[15] de Souza A.C.Z., Castilla M. (2019).

[16] Krommydas K.F., Alexandridis A.T. (2020). Nonlinear analysis methods applied on grid-connected photovoltaic systems driven by power electronic converters. IEEE Journal of Emerging and Selected Topics in Power Electronics.

[17] Babaiahgari B., Jeong Y., Park J.-D. (2021). Dynamic control of region of attraction using variable inductor for stabilizing dc microgrids with constant power loads. IEEE Transactions on Industrial Electronics.

[18] Kumar S., Islam S., Jolfaei A. (2019). ’microgrid communications - protocols and standards. Variability, Scalability and Stability of Microgrids.

